# Physical Exercise as a Diagnostic, Rehabilitation, and Preventive Tool: Influence on Neuroplasticity and Motor Recovery after Stroke

**DOI:** 10.1155/2015/608581

**Published:** 2015-11-23

**Authors:** Caroline Pin-Barre, Jérôme Laurin

**Affiliations:** ^1^Aix Marseille Université, CNRS, ISM UMR 7287, 13288 Marseille, France; ^2^Université de Nice Sophia-Antipolis et Université de Sud Toulon-Var, LAMHESS, UPRES EA 6309, 06204 Nice, France

## Abstract

Stroke remains a leading cause of adult motor disabilities in the world and accounts for the greatest number of hospitalizations for neurological disease. Stroke treatments/therapies need to promote neuroplasticity to improve motor function. Physical exercise is considered as a major candidate for ultimately promoting neural plasticity and could be used for different purposes in human and animal experiments. First, acute exercise could be used as a diagnostic tool to understand new neural mechanisms underlying stroke physiopathology. Indeed, better knowledge of stroke mechanisms that affect movements is crucial for enhancing treatment/rehabilitation effectiveness. Secondly, it is well established that physical exercise training is advised as an effective rehabilitation tool. Indeed, it reduces inflammatory processes and apoptotic marker expression, promotes brain angiogenesis and expression of some growth factors, and improves the activation of affected muscles during exercise. Nevertheless, exercise training might also aggravate sensorimotor deficits and brain injury depending on the chosen exercise parameters. For the last few years, physical training has been combined with pharmacological treatments to accentuate and/or accelerate beneficial neural and motor effects. Finally, physical exercise might also be considered as a major nonpharmacological preventive strategy that provides neuroprotective effects reducing adverse effects of brain ischemia. Therefore, prestroke regular physical activity may also decrease the motor outcome severity of stroke.

## 1. Introduction

Despite progress in functional rehabilitation, stroke patients frequently present chronic motor disabilities [1]. Therefore, it seems crucial that both scientists and therapists continue to investigate stroke physiopathology and improve the effectiveness of physical training programs on motor recovery. In recent years, physical exercise was used in stroke experiments for 3 main purposes, namely, the detection of physical dysfunctions, the improvement of motor activity, and the prevention of severe damage. Such contributions might ultimately improve both physical independence and quality of life, while reducing cardiovascular complications and recurrent stroke [2, 3].

First, acute fatiguing exercise is used in preclinical experiments as a diagnostic tool to detect sensorimotor dysfunctions and/or reveal treatment effectiveness that cannot be observed in resting condition. Indeed, alteration of motor unit activation in both affected and unaffected sides or changes of the motor reflex regulation were highlighted during/after acute exercise [4, 5]. Moreover, a higher corticospinal tract activity was found only after acute treadmill exercise in trained patients contrary to untrained patients [6].

Then, added to its beneficial effects on cardiorespiratory fitness and muscular endurance, chronic physical activity is effective as a rehabilitation tool for improving functional recovery and promoting neural plasticity [3, 7]. Indeed, it was recently found that physical training promoted cerebral angiogenesis, vasomotor reactivity, and neurotrophic factor release but also reduced apoptosis processes, excitotoxicity, and inflammation into the peri-infarct site and could improve the regulation of motor unit activation [8–12].

Nevertheless, physical training seems to remain insufficient to completely restore neural and motor functions. A recent approach for stroke therapy is to combine physical training with pharmacological treatments, known to accentuate and/or accelerate neuroplasticity. In the present review, we discuss the influence of physical training with or without additional pharmacological treatment on neuroplasticity and motor recovery after stroke.

Although regular exercise reduces the risk of developing stroke [13], it may occur for physically active individuals [14, 15]. However, endogenous neuroprotective effects induced by prestroke physical activity may exhibit beneficial influence on recovery by reducing both brain damage and motor outcome severity [16–19].

The present review is designed to discuss the multiple uses of physical exercise to improve neural and motor recovery following cerebral ischemia/stroke from both animal and human studies. We suggested that exercise-induced neural plasticity is crucial to better understanding motor outcomes after stroke and improving rehabilitation program effectiveness.

## 2. Acute Exercise-Induced Neural Adjustments after Stroke

Animal and human studies reported that acute exercise could reveal changes in muscle activation regulation after cerebral ischemia. More precisely, the electromyographic (EMG) activity was lower in affected hindlimb muscles during a single bout of treadmill exercise compared to unaffected muscles, reflecting a strong decrease of the motoneuronal recruitment from spinal and/or supraspinal motor pathways [4, 20]. A reduced corticospinal excitability to the paretic quadriceps was also observed by showing strong decrease of the motor evoked potential (MEP) amplitude during a running exercise [4]. In addition, cortical activation increased in the unaffected side while it decreased in the affected side after ankle dorsiflexion movements that suggested compensatory neural mechanisms [21–26].

Spinal motor reflex plasticity also seems to contribute to motor disorders after stroke but the underlying neural mechanisms remain poorly understood [36, 37]. A recent study has demonstrated that the motor reflex regulation (H reflex) was acutely altered following an exhaustive isometric exercise on affected triceps brachii in MCAO rats whereas resting H reflex did not differ between injured and noninjured animals [5]. It was postulated that such findings might be partially due to an alteration of motor reflex regulation by muscle afferents that remained activated after intense exercise (groups III and IV muscle afferents).

Fatigue process alterations could also be observed during dynamic exercise after cerebral ischemia by using EMG recording. When fatigue progresses, there is a shift to lower motor unit frequencies. Median power frequency (MPF), which is the sum of product of the EMG power spectrum and the frequency divided by the total sum of the power spectrum, is frequently used for the assessment of muscle fatigue in surface EMG signals [38]. Therefore, a decrease in MPF serves as an index of fatigue [39]. After cerebral ischemia, the observed MPF drop at the unaffected hindlimb reflected a higher muscle fatigue compared to the affected one (no MPF decrement). A lesser fatigue-related decrease in median frequency was also observed in humans at the paretic side compared to the nonparetic side during both voluntary contractions and locomotor activity [20, 40]. This accelerated fatigue process of the unaffected hindlimb was usually explained by its higher weight bearing that compensated for the affected side. Nevertheless, reduction of work without MPF decrease of affected muscle was reported during repeated eccentric-concentric actions suggesting that peripheral fatigue through excitation-contraction coupling disruption might contribute to exercise intolerance and neuromuscular disorders of stroke patients [41].

It was also found that acute exercise-induced neural adjustments could reveal the effectiveness of a given treatment. Indeed, Forrester and colleagues have first compared trained and untrained stroke patients on the observed neural response to a single bout of treadmill exercise [6]. They found that the MEP amplitude increased in trained patients after this exercise contrary to untrained individuals, in which MEP amplitude remained unchanged. Such training corticospinal adaptation was not observed in the preexercise MEP amplitudes, but only after exercise, and provided insight into the effectiveness of 6-month treadmill training [6].

## 3. Effects of Poststroke Physical Training on Neuroplasticity and Motor Recovery

**Figure 1:**
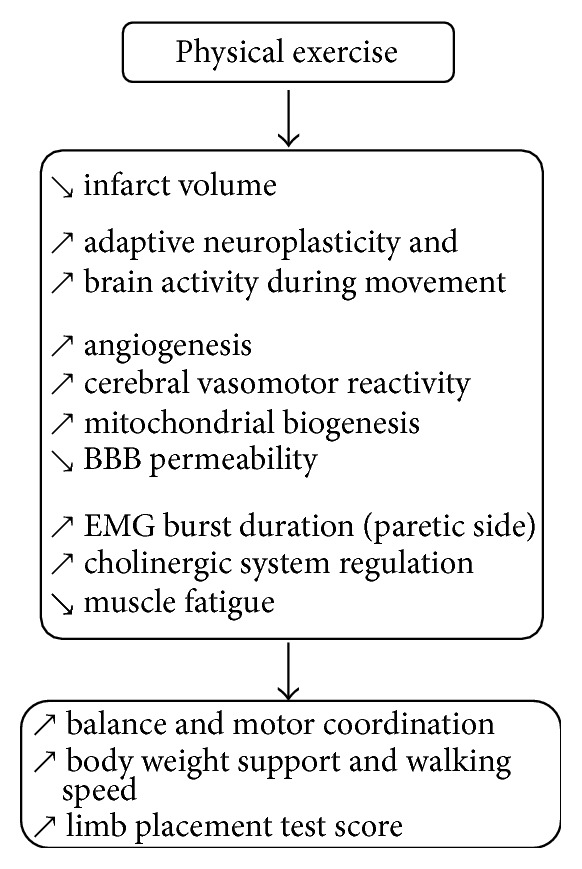
Beneficial structural and functional plasticity induced by physical exercise in stroke individuals.

The effects of chronic physical training in stroke patients have received more attention in scientific literature than the ones of acute exercise. Indeed, physical rehabilitation remains the first-line intervention strategy to attenuate chronic impairments of sensorimotor function by promoting brain organization and reducing infarct volume during the first weeks after stroke [42, 43]. Given that the infarct size was not always correlated with motor recovery, it was suggested that other adaptive mechanisms might take part [44, 45]. Numerous recent studies indicated that early treadmill training promotes neuroplasticity by acting on brain vasomotor activity and angiogenesis, neurotrophic factor and apoptosis marker expressions, brain inflammatory processes, blood brain barrier (BBB) integrity, and muscle activation control (Figure 1) [46–48].

Cerebral blood flow in the ischemic region is affected following human stroke and cerebral ischemia in mice due to impaired cerebral vasomotor reactivity [8, 49]. Therefore, restoring an adequate cerebral vasomotor reserve capacity is crucial to supplying required nutriments and O_2_, as well as reducing infarct volume and functional deficits [50, 51]. Preclinical and clinical studies bring strong evidence on endurance training effectiveness to promote vasomotor reactivity (endothelium-dependent vasorelaxation) and angiogenesis in the ischemic penumbra, which contribute to limiting brain damage and motor deficits [9, 50]. Indeed, treadmill training increased the most commonly studied angiogenic growth factor expression, namely, vascular endothelial growth factor (VEGF), and its regulatory protein, caveolin-1, that might contribute to explaining the increase of new vessel growth and vascular density [52–56]. The training effect on angiogenesis was reinforced by evidence showing that 2 weeks of treadmill training increased the angiopoietin expression, another angiogenic growth factor that has a key role in new vessel formation [54]. Other findings revealed that the area of platelet-endothelial cell adhesion molecule- (PECAM-1-) immunopositive cells (protein involved in angiogenesis and integrin activation) was significantly increased around the infarct after 28 days of treadmill training [46]. Daily treadmill training induced an increase of GFAP expression (proteins playing a role in vascular cerebral plasticity) suggesting that area of neovascularization was higher in exercised animals [47]. Moreover, voluntary running training upregulated by 3- to 4-fold aortic endothelial nitric oxide synthase (eNOS) mRNA expression and it remained elevated 10 days after training [8, 9]. Such findings concur with the fact that beneficial effect of running was completely abolished in animals lacking eNOS expression and in mice treated with a NOS inhibitor or an antiangiogenic compound such as endostatin [9]. Moreover, training program increased endothelial progenitor cells (EPC) in bone marrow that are known to strongly influence eNOS expression. It is noteworthy to add that only 3 days of aerobic exercise reduced brain microvascular endothelial cell apoptosis related to the increase of shear stress that was followed by modest improvement of cerebral blood flow [51].

Physical training can also upregulate the expression of some neurotrophic factors, such as brain-derived neurotrophic factor (BDNF), nerve growth factor (NGF), and insulin-like growth factor (IGF-1).

BDNF is one of the most active neurotrophins that binds to the tyrosine kinase receptor (trkB). Overall, this association triggers several molecular pathways that promote neural proliferation and survival and synaptic and axonal plasticity by enhancing synapse formation, dendritic growth, and remodeling [57]. Furthermore, BDNF seems also to act on motor function because blocking BDNF mRNA expression by injecting antagonist reduced skilled motor abilities [58].

The neuroprotective effect of endogenous and exogenous BDNF is well established after brain infarction [12, 59–62]. Basal endogenous BDNF/trkB expression increased within both hemispheres following moderate cerebral ischemia (including the penumbra) during the first 2 weeks but motor recovery remains widely insufficient [63–65]. Therefore, treatments that enhance BDNF/trkB production seem relevant to improve motor recovery (even if infarct size is not modified) [11, 66]. It was found that delayed intravenous BDNF administration (20 *μ*g/day) for 5 days improved long-term functional outcomes such as running and sensorimotor recovery after ischemia [11, 66, 67].

Interestingly, endurance training stimulated endogenous BDNF/tkrB expression and may play a neuroplastic role following cerebral ischemia or intracerebral hemorrhage in rat and mice [58, 63, 68–72]. This enhanced BDNF/tkrB was observed in both hemispheres but especially in the nonlesional hemisphere compared to healthy animals under the same program (that might represent a compensatory mechanism) [53, 69]. Despite the fact that there is no direct evidence, the training-related BDNF expression is often associated with motor recovery improvement [58, 63, 70]. It was demonstrated that 7 consecutive training days could be sufficient to increase BDNF expression and improve motor recovery [72–74]. Although endurance exercise upregulates BDNF expression in striatum and cortex, functional outcome improvement might be more related to hippocampal BDNF expression [70, 72]. Indeed, voluntary wheel exercise is known to induce substantial motor recovery associated with the highest hippocampal BDNF level [70]. Another study also found a positive correlation between motor function recovery rate and hippocampal BDNF expression after treadmill training following cerebral infarction [72].

Finally, the number of NGF-immunopositive cells, promoting cell growth and neuronal activity, was particularly increased over a widespread region around the infarct zone in trained rats [46]. NGF expression, which may be the result of heightened neuronal activity during exercise, could contribute to reducing brain damage around 4 weeks after ischemic stroke.

Physical training could reduce ischemic brain damage and motor deficits by other mechanisms such as reduction of acute inflammatory reactions and neuronal apoptosis [75]. Indeed, it was demonstrated that the neurotrophic factor midkine (MK), which could delay the process of neuronal death during the early phase after cerebral infarction, was higher expressed in the cells of the peri-infarct region after physical training program [46].

Cerebral ischemia-induced cell death is commonly attributed to necrosis and apoptosis, but it was recently found that inappropriate autophagy might also lead to cell death [76]. However, physical training mitigated autophagosomes accumulation and attenuated apoptosis in the peri-infarct region while improving the modified neurological severity score scale. Physical training could also upregulate IGF-1 expression, which is known to attenuate autophagy and also to promote neurogenesis [75].

In addition, several studies reported that neuronal death in both the striatum and the cerebral cortex caused a degeneration of nigral dopaminergic neurons that are known to contribute to regulating motor activity [77]. However, treadmill training promoted axon regeneration of newborn striatonigral and corticonigral projection neurons in ischemic brain while improving motor function. It was thus suggested that exercise could enhance restoration of functional neural circuitry within the basal ganglia. The cerebellum plays an important role in the motor coordination, learning, and equilibrium and also seems to be involved in motor deficits following cerebral injury. Therefore, it is not surprising that treadmill training promoted both synaptogenesis and neurite outgrowth via 25-kDa synaptosomal-associated protein expression in the cerebellum. Likewise, such training also increased glial fibrillary acidic protein in the cerebellum, which plays a role in axonal growth, and improved motor coordination as observed with the rotarod test (longer time to fall from the rotating rod) [47].

Disruption of the BBB, increasing thus its permeability, is one of the major contributors to the pathogenesis of cerebral ischemia. Such event was observed when matrix metalloproteinase-9 (MMP-9) is strongly upregulated that impairs the extracellular matrix of the BBB [78, 79]. However, physical training attenuated BBB disruption as revealed by a decrease of MMP-9 expression and a parallel increase expression of MMP-9 inhibitor, the tissue inhibitor of metalloproteinase-1 (TIMP-1). Interestingly, such mechanisms might contribute to decreasing the observed neurological deficits, infarct volume, and brain edema [80].

These neuroplasticity processes require energy from mitochondria. However, cerebral ischemia induces damage of the cerebral mitochondria biogenesis, contributing to the extent of neuronal ischemic injury [81]. Nevertheless, 7-day endurance training increased mitochondrial biogenesis after cerebral ischemia by enhancing both the amount of mitochondrial DNA and the expression of numerous mitochondrial biogenesis factors such as the mitochondrial transcription factor proliferator activated receptor coactivator-1 (PGC-1), nuclear respiratory factor 1 (NRF-1) protein, and mitochondrial transcription factor A (TFAM) [82]. It was suggested that these events are involved in decreasing infarct volume and the improvement of neurological score.

Very few studies using rat model of cerebral ischemia enable better understanding of the treadmill training outcomes on muscle activation regulation. Cerebral ischemia is believed to affect synaptic activity including the cholinergic system, which leads to decreasing both neuromuscular junction and cholinergic brain synaptic transmission. It was shown that aerobic treadmill training (20 min/day, during 21 days) improved cholinergic system regulation/homeostasis by decreasing acetylcholinesterase activity (i.e., hydrolyzing the acetylcholine) and by enhancing choline acetyltransferase activity (i.e., synthesis of acetylcholine) [53]. It was thus suggested that such adaption might allow better motor limb function as indicated by improvement of the limb placement test score. Moreover, 10 days of treadmill training improved balance, functional outcome (behavioral score), and motor coordination. Indeed, the asymmetry of muscle activation pattern between affected and unaffected hindlimbs was restored because the EMG burst duration was increased at the affected side [83]. Muscle fatigue observed during locomotion was reduced as indicated by the increase of MPF at the unaffected side (see Section 2) [4].

A previous human study indicated that effective gait training on treadmill with body weight support, improving walking speed and endurance, was characterized by an increase of brain activity in the bilateral primary sensorimotor cortices, the cingulate motor areas, the caudate nuclei bilaterally, and the thalamus of the affected hemisphere during paretic foot movement [84]. In addition, treadmill exercise (improving cardiovascular fitness by 18%) activates subcortical neural networks during single knee movements of the lower extremity, as observed by fMRI. Indeed, physical training changed brain activation during paretic limb movement, showing 72% and 18% increased activation in posterior cerebellar lobe and midbrain, respectively [85]. After 10 weeks of training, the improvement in sensorimotor function, assessed with the Fugl-Meyer Index, seemed strongly related to the improvement in aerobic capacity [86].

It is noteworthy that some neural mechanisms after physical exercise remain to be investigated such as diaschisis. Indeed, functional deficits may also be associated with distant effects after subcortical lesions resulting from deafferentation to a region not directly involved in the stroke. Moreover, it was found that several rehabilitation exercises, inducing blood flow and metabolic changes in the contralesional hemisphere, might act on diaschisis. Given that alleviation of diaschisis could contribute to motor recovery [87], it seems important that further studies will determine how endurance exercises may influence diaschisis after cerebral ischemia.

## 4. Influence of Exercise Parameters on Neuroplasticity and Functional Outcomes

Depending on the chosen exercise parameters (volume, intensity, session frequency, and timing of exercise initiation), neuroplasticity can be either adaptive or maladaptive to recovery and, thus, may affect the training effectiveness after cerebral ischemia [46, 87–91]. Strong evidence suggested a time-limited period of enhanced neuroplasticity [89, 92–94]. Animal studies underlined that the limb function was less improved when training started before 24 h after ischemia compared with a start during the 5 first days [89]. Moreover, early intense training might induce larger cortical infarct volume and thalamic atrophy when the program starts before 24 h [95]. Exercise detrimental effects were also observed on neuroplasticity when animals performed treadmill exercise shortly after trauma [96]. The lesional aggravation might be related to localized and prolonged hyperthermia. Indeed, physical exercise, known to induce hyperthermia, could accentuate the cerebral ischemia-induced release of glutamate and catecholamines that lead to neural excitotoxicity [95, 97]. However, it seems important to add that other processes than hyperthermia might be involved in the sensorimotor deficit aggravations and need to be explored. Furthermore, early running training downregulated proteins involved in neuroplasticity such as MAPK, CAMKII, PKC, synapsin I, or CREB expression [98] and decreased the level of proinflammatory cytokines known to be related to neuroprotection [99, 100]. Finally, Yagita et al. (2006) had shown that two weeks of running exercise reduced the number of newborn neurons in ischemic rats and thus limited neurogenesis in the hippocampus [101]. The author suggested that running was too stressful and enhanced the corticosterone level, known to decrease neurogenesis. Likewise, an immediate overuse of the lesioned forelimb could also increase tissue loss around the lesion and aggravated sensorimotor deficits on a longer term [102–104]. One explanation could be related to anatomical damage resulting from the reduction of dendritic growth in the ischemic hemisphere [104].

In addition, an augmented volume of exercise or increase of inpatient therapy duration for stroke did not indicate superior effects on functional recovery and activities of daily living [105, 106]. Interestingly, when healthy active men were subjected to a strong increase of volume training, physical performance was also not improved [107]. It thus seems that volume is not the main exercise parameter that should be investigated for improving aerobic program.

Greater improvements were observed with higher exercise intensities after stroke and neurodegenerative disease but also for healthy individuals [92, 108–110]. However, the effect of intensity on neuroplasticity remains unclear because it is poorly investigated in stroke patients or in ischemic animal models (ongoing study in our laboratory). It should also be pointed that one major methodological limitation is related to the exercise intensity determination. Indeed, training intensity was mainly based on maximal oxygen consumption or maximal exercise heart rate (or even intensity based on empirical values). However, these parameters were not appropriated because stroke patients never reached maximal capabilities. It was recently suggested that prescribing intensity should rather be based on submaximal parameters such as ventilatory or lactic threshold that were more accurate in distinguishing moderate to intense exercise [111].

## 5. Beneficial Effect of Physical Training Rehabilitation in Combination with Pharmacological Treatments

Using multiple recovery processes (physical exercise and pharmacologic treatments) may be critical for enhancing functional outcomes, in contrast to monotherapies targeting single mechanisms (Table 1).

Given that recent findings have established that anti-inflammation may be an important target for stroke treatment [112], some authors demonstrated that combination of skilled reaching training with indomethacin or minocycline accentuated recovery [10]. First, beneficial effects of such combination included an improvement of sensorimotor function as indicated by higher correct placements of the impaired forelimb during a walking task. Secondly, the number of proliferating microglia was more reduced and the survival of newborn astrocytes in the peri-infarct zone was increased compared to rats that underwent training alone. Authors suggested that the motor outcome improvement might be partially explained by the observed changes of neural response.

In addition, therapeutic drug such as S-nitrosoglutathione (GSNO) exhibits similar neurovascular protecting effects compared to physical training following traumatic brain trauma and cerebral ischemia in rats [18, 27]. Administration of GSNO could reduce neuronal apoptotic cell death, excitotoxicity, and inflammation as well as protecting BBB integrity. GSNO could also stimulate the expression of VEGF and BDNF after traumatic brain injury. Several authors have demonstrated that combining rotating rod motor exercise with GSNO administration accelerated and accentuated both walking and balance abilities compared to the effects induced by each treatment applied separately. Moreover, the improved motor function was associated with a reduction of both infarct volume and neuronal cell death as well as an increase of PECAM-1 and BDNF expression [28].

D-amphetamine, acting primarily through norepinephrine and dopamine mechanisms, is a potent modulator of neurological function and cortical excitation that facilitated motor skill abilities [113]. It was demonstrated that a single injection of D-amphetamine on the first day of training facilitates effectiveness of motor skill training compared with D-amphetamine treatment alone after a focal cortical infarct in squirrel monkeys [29]. However, administration of a high dose of D-amphetamine combined with rehabilitation training failed to promote fine motor recovery in a rat embolic stroke model. Authors explained this result by the fact that the dose of D-amphetamine was too high, thereby limiting animal engagement in the staircase test [114].

Other pharmacological agents could improve motor recovery by focusing on different neural mechanisms. For example, Nogo-A protein, a myelin-associated inhibitor, appears to be partially responsible for inhibition of axonal growth in white matter. Therefore, suppressing this myelin-associated neurite outgrowth inhibitor by specific antagonists of Nogo-A, such as NEP 1-40 or NGR(310)Ecto-FC, increased neurite outgrowth and axonal regeneration after stroke [30, 31]. Five-week motor training combined with NEP 1-40 treatment accelerated motor recovery (skilled reach and foot fault tests) from the first week after cerebral ischemia compared to treatments applied alone in which beneficial effects were observed later (weeks 2 and 4) [30]. Unfortunately, neural plasticity mechanisms underlying this improvement remain unknown, but these findings showed that this therapy combination could accelerate the recovery process following cerebral ischemia.

Neurons and glial cells in brain can synthesize progesterone that exhibits neuroprotective effects after cerebral ischemia [115–118]. Rehabilitation could increase reorganization of cortical maps whereas the progesterone might reduce the infarct volume through its neuroprotective effect by targeting excitotoxicity. Although this combination had no additional effect in reducing infarct volume, functional recovery effectiveness was promoted as shown by the increased rotarod performance and forelimb grip strength at all time points within 7 days after ischemic stroke [32].

In addition, some authors indicated that combination of two pharmacological treatments could improve functional recovery and neural plasticity compared to either treatment alone. For example, association of epidermal growth factor (EGF) and erythropoietin (EPO) treatments is more effective in promoting tissue regeneration and proliferation of neural precursor cells than monotherapy [33]. However, an even greater improvement in skilled reaching ability in the staircase test was observed when physical rehabilitation was added to serial application of both EGF and EPO [34]. Authors have also indicated that fine motor skill improvement was observed 10 weeks after functional rehabilitation alone whereas 4-week-long treatment combination was sufficient to improve sensorimotor function. It means that functional recovery is also clearly accelerated with this combination.

Another strategy consists of recreating a tissue environment free from growth inhibiting molecules. Chondroitinase ABC, which degrades inhibitory chondroitin sulphate proteoglycans present in the extracellular matrix, reduces the neurite-inhibitory environment and facilitates axonal sprouting and sensorimotor recovery after spinal cord injury [119, 120] and after cerebral ischemia [121, 122]. This treatment was applied at the ipsilesional cortex in rats in combination with functional rehabilitation. Synaptic plasticity, assessed by measuring the expression of glutamate vesicular transporter (vGLUT1 and vGLUT2) and the GABA vesicular transporter (vGAT), and functional recovery were promoted by the synergic effect of these treatments [35].

Given that some promising treatments developed in preclinical studies failed to be effective in clinical studies [123, 124], it seems crucial to assess the pharmacological effectiveness in stroke patients before combining it with physical exercise. Therefore, we need to keep in mind that not all the effective combinations found in animal studies could be considered effective for human. Nevertheless, assessing both treatments and exercise in rodent models seems relevant to found new therapeutic strategies to highlight the most effective and applicable strategies for human.

## 6. Influence of Prestroke Physical Activity on Motor Dysfunction Severity

To avoid administration of several pharmacological substances in individuals with high risks of stroke (may induce deleterious interaction between drugs and complications due to their side effects), physical activity might be a major alternative preventive strategy for reducing the brain secondary injury when stroke occurs. Several recent human and animal studies have well demonstrated that preischemic physical activity may reduce initial stroke severity on functional motor outcomes, edema, and infarct volume by acting on inflammation, vascular processes, BDNF expression, and metabolic disorders [14, 16, 17, 125, 126].

Ischemia-induced brain inflammation is believed to play a pivotal role in the development of secondary brain injury by intensifying the inflammatory cell accumulation and microvascular impairments. Indeed, adhesion molecules such as intercellular adhesion molecule 1 (ICAM-1) promote leukocyte infiltration into injury site and leukocyte adhesion to microvascular endothelium. Preischemic physical training-induced neuroprotection was associated with a decrease in ICAM-1 mRNA expression and in the number of ICAM-1-positive vessels [17]. Consequently, leukocyte accumulation in damaged cortex and striatum vessels decreased, resulting in reduction of brain inflammation during reperfusion.

Brain angiogenesis is another neuroprotective mechanism for reducing the detrimental motor effect of cerebral ischemia. Physical training prior to cerebral ischemia increased short- and long-term effects on microvascular density and cerebral blood flow by promoting angiogenesis and increasing cerebral vasomotor reactivity. It was found that preischemic exercise improved vasorelaxation by enhancing VEGF levels into the ischemic region, which is known to activate eNOS and EPCs recruitment [8, 19]. Likewise, exercise decreased the endothelin-1 (a powerful vasoconstrictor agent) expression, thereby reducing the impact of vasoconstriction on cerebral blood flow [127].

Preischemic training could also attenuate brain damage by limiting metabolic disorders after cerebral ischemia. Indeed, 5′AMP-activated protein kinase (AMPK), phosphofructokinase-1 (PFK), and hypoxia-induced factor-1*α* (HIF-1*α*), involved in glycolysis [18, 128], were significantly higher in preischemic trained rats [129]. Moreover, the increase of glucose transporters in neurons and endothelial cells of the BBB (GLUT3 and GLUT1, resp.) results in an improved glucose oxidation immediately after cerebral ischemia, allowing a faster and more substantial increase in ATP production.

Moreover, it was postulated that preischemic exercise (30 min on a treadmill, 5 days/week for 3 weeks) decreases BBB dysfunctions and, thus, reduces infarct volume and edema as confirmed by the MMP-9 expression decrement [130, 131]. Preischemic treadmill training-induced neuroprotection in ischemic rats increased endogenous BDNF expression that leads to motor recovery improvement [16, 17, 67, 132]. Interestingly, the preischemic treadmill exercise improved the therapeutic effectiveness of postischemic treadmill training on motor function compared with animals performing it only after cerebral ischemia [53].

Neuronal excitotoxicity induced by excessive glutamate release is a major deleterious event of the brain secondary injury [133, 134]. It was found that preischemic treadmill training could also reduce the expression of glutamate receptors, mGluR5 and NR2B, reflecting a decrease of glutamate effect on surrounding cells [135–137]. Moreover, it was postulated that the reduction of infarct volume induced by 12 weeks of treadmill might be related to increase of NGF expression and this receptor, p75, known to promote neuroprotection against excitotoxicity and free-radical damage [138].

Individuals who were physically active prior to stroke have been shown to exhibit less deleterious functional outcomes, as indicated by higher Barthel Index scores as well as Oxford Handicap Scale [14, 15]. Indeed, it was recently revealed that patients with high levels of prestroke physical activity were associated with milder stroke severity at admission, with faster early motor improvement and with a lower final infarct volume [139]. Interestingly, it seems that simply walking 1 h/day during 5 days per week or doing a vigorous aerobic activity 1 h/day twice a week is sufficient to induce preventive effects. However, findings remain conflicting in human studies because no strong association between higher levels of physical activity and better functional outcomes after stroke was found in a large prospective cohort [126].

## 7. Conclusion

Although acute exercise appears to be useful for better understanding neural and motor recovery mechanisms, few studies have used this experimental model to investigate stroke physiopathology. Neural adaptations remain thus unclear. Furthermore, physical training appears to be promising to improve functional motor recovery by promoting neuroplasticity at different cellular and molecular levels. These beneficial effects seem accelerated and/or accentuated when exercise is combined with an additional pharmacological treatment. Nevertheless, optimal parameters of training and treatment need to be investigated to maximize/accelerate neuroplasticity and motor recovery and avoid undesirable effects of exercise. All these findings could help researchers and therapists to justify the effectiveness of their physical training programs in order to increase the patient willingness to regularly perform physical activity before and after stroke.

## Figures and Tables

**Table 1:** Influence of pharmacological agents associated with physical exercise on motor recovery after brain stroke.

Drug agents	Targets	Results	References
Indomethacin Minocycline	Inflammatory processes	↘ infarct volume (indomethacin only) ↗ sensorimotor performance ↘ microglia ↗ astroglia	[10]

GSNO	Oxidative stress Inflammatory processes Excitotoxicity	↘ infarct volume, apoptotic cell death ↗ neurological score, motor recovery, and survival rate ↗ CBF, synaptic plasticity, and BBB leakage ↘ TNF-*α*, IL-1*β*, and iNOS	[18, 27, 28]

D-Amphetamine	Noradrenergic *α*1-receptor agonist	↗ motor recovery	[29]

NEP 1-40	Nogo-A protein inhibitor	↗ early motor recovery ↗ axonal growth	[30]

NgR(310)Ecto-Fc	Nogo-NgR pathway inhibitor	↗ motor recovery ↗ axonal plasticity	[31]

Progesterone	Excitotoxicity Inflammatory processes	↘ infarct volume ↗ forelimbs strength and motor recovery	[32]

EGF^*∗*^ and EPO^*∗*^	Neuron proliferation, migration, and differentiation	↗ accelerated fine motor recovery	[33, 34]

Chondroitinase ABC	Chondroitin sulphate proteoglycans (CSPGs)	↘ CSPGs ↗ synaptic plasticity ↗ motor recovery	[35]

*↗* indicates an increase and *↘* a decrease, respectively; NEP 1-40: NOGO extracellular peptide; EGF: epidermal growth factor; EPO: erythropoietin; GSNO: S-nitrosoglutathione; CBF: cerebral blood flow; BDNF: brain developed neurotrophic factor; trkB: tropomyosin receptor kinase B; BBB: blood brain barrier. ^*∗*^Molecules combination.
